# Marmosets as models of infectious diseases

**DOI:** 10.3389/fcimb.2024.1340017

**Published:** 2024-02-23

**Authors:** Ian C. T. Herron, Thomas R. Laws, Michelle Nelson

**Affiliations:** CBR Division, Defence Science and Technology Laboratory (Dstl), Salisbury, United Kingdom

**Keywords:** common marmoset, immunology, inflammation, animal models, *Francisella tularensis*, *Burkholderia pseudomallei*, hepatitis C virus

## Abstract

Animal models of infectious disease often serve a crucial purpose in obtaining licensure of therapeutics and medical countermeasures, particularly in situations where human trials are not feasible, i.e., for those diseases that occur infrequently in the human population. The common marmoset (*Callithrix jacchus*), a Neotropical new-world (platyrrhines) non-human primate, has gained increasing attention as an animal model for a number of diseases given its small size, availability and evolutionary proximity to humans. This review aims to (i) discuss the pros and cons of the common marmoset as an animal model by providing a brief snapshot of how marmosets are currently utilized in biomedical research, (ii) summarize and evaluate relevant aspects of the marmoset immune system to the study of infectious diseases, (iii) provide a historical backdrop, outlining the significance of infectious diseases and the importance of developing reliable animal models to test novel therapeutics, and (iv) provide a summary of infectious diseases for which a marmoset model exists, followed by an in-depth discussion of the marmoset models of two studied bacterial infectious diseases (tularemia and melioidosis) and one viral infectious disease (viral hepatitis C).

## 1 Introduction

### 1.1 The common marmoset (*Callithrix jacchus*)

The common marmoset (*Callithrix jacchus*), henceforth referred to as the marmoset, is a Neotropical new-world (now increasingly referred to as platyrrhines) non-human primate (NHP) native to the north-eastern regions of Brazil. Having diverged from humans some 33 million years ago, the common marmoset is phylogenetically and anatomically more similar to humans than rats or mice which diverged approximately 96 million years ago. As such, a significant degree of cross-reactivity of reagents designed for human targets with those in the marmoset is observed (Barton et al., 1984; Neubert et al., 1996; Kireta et al., 2005; Jagessar et al., 2013; Neumann et al., 2016). However, a greater evolutionary distance exists between the divergence of new-world NHPs from humans compared with old-world (now increasingly referred to as catarrhines) NHPs (e.g. rhesus macaques and cynomolgus macaques) from humans, with the latter occurring some 23 million years ago (Mansfield, 2003). Consequently, there exists more physiological and immunological differences between humans and marmosets than humans and old-world primates, which have traditionally been used as NHP models of various human diseases. Nevertheless, the marmoset represents an attractive alternative to the old-world primates and this is reflected by their increasing use in the field of biomedical science.

Whilst a comprehensive review of the basic biology and physiology of the marmoset is beyond the scope of this manuscript, the reader is directed to a number of excellent reviews published on these topics (Abbott et al., 2003; Orsi et al., 2011; ‘T Hart et al., 2012; Preuss, 2019). Here, a brief overview of the marmoset is presented to provide the reader with sufficient background to appreciate the pros and cons of using this new-world primate as a model of infectious diseases. This information is also summarized in **Table 1**. The marmoset is considerably smaller than the old-world primates, weighing around 350 to 450 g with a body size comparable to that of a rat (Orsi et al., 2011). As such, animals are more easily handled and the associated costs (e.g., husbandry, housing, feeding, etc.) are reduced considerably. Additionally, their smaller size makes biocontainment both safer and cheaper. The small size of the marmoset means smaller amounts of a given test substance/therapeutic can be administered, again reducing costs and aiding where manufacture is difficult. Aside from their small size, marmosets have a compact life-span and reach sexual maturity in approximately 1.5 years. Marmosets are easily bred in captivity and frequently give birth to multiple offspring; these offspring are born as bone marrow chimeric twins that are the result of fusion of the placental bloodstreams (Benirschke et al., 1962; Sweeney et al., 2012). Consequently, marmoset twins are immunologically highly comparable. In this regard, the marmoset is biologically unique; researchers can exploit this aspect of their biology to perform paired experimental analyses, i.e., where one sibling receives treatment with a given therapeutic and the other receives a placebo. Such paired analyses are highly beneficial, particularly in pre-clinical studies. Further, marmoset twins have been used in adoptive transfer experiments in the study of the pathogenesis of multiple sclerosis (MS) (Massacesi et al., 1995; Genain and Hauser, 1997). Importantly, marmosets are a naturally outbred species and are exposed to environmental factors (e.g., bacteria) that shape their developing immune systems. As the links between the environmental microbiome and host immune system continue to emerge, this feature of the marmoset is particularly advantageous as it better reflects the human condition. Marmosets are susceptible to infection with many wild-type viruses that, in their native forms, either do not cause disease or cause a different disease in the mouse (Mansfield, 2003; Carrion and Patterson, 2012). Indeed, to render mice vulnerable to infection, an adapted rodent virus is frequently used. These viruses, although based on the wild-type virus, are genetically modified and thus may fail to recapitulate human disease (Sarkar and Heise, 2019). Finally, and of particular importance to infection models, marmosets are not known to carry endogenous viruses that cause disease in humans (Abbott et al., 2003). Thus, with fewer biosafety considerations the marmoset represents an animal model that is safer, cheaper and less labor intensive.

**Table 1:** Advantages and disadvantages of the common marmoset as a small animal model of disease.

Advantages	Disadvantages
Small size (approximately 350 to 450 g)	Limited blood draw volumes
Compact life-span	Increased cost/gestation period (compared to rodents)
Cheaper to house and feed/lower husbandry costs	No germ-free marmosets
Early sexual maturity and high reproductive efficacy (multiple offspring)	Studies restricted to smaller numbers of animals
Susceptible to infection with wild-type viruses	Fewer analytical tools (immunological/molecular, etc.) available
Disease closely mimics human disease	Ethical concerns of using NHPs
Fewer biosafety concerns (free from endogenous organisms that cause disease in humans)	Increased evolutionary distance from humans compared with old-world primates, e.g., rhesus macaque and cynomolgus macaque
Easier and safer to contain in biocontainment	
Immunological repertoire very similar to that in humans (~86% identical between marmoset and human)	
Many human reagents are cross-reactive with marmoset	

Whilst the marmoset presents a number of practical advantages, it is vital that the potential disadvantages of the species are not overlooked. For example, though the marmoset is comparatively cheaper and easier to handle than the larger old-world primates, mice are both considerably smaller and cheaper than the marmoset. Whilst the small size of the marmoset may be advantageous, this may also limit what procedures/techniques can be performed. For example, the amount of blood that can be obtained from a live marmoset is typically 1% of its body weight (Jagessar et al., 2013; ‘T Hart, 2019). A study wishing to perform comprehensive immunophenotyping of marmoset immune cells may not be feasible given the limited amount of blood available at each blood draw – particularly those studies incorporating large panels that require multiple controls. While outbred animals are more representative to humans, this heterogeneity may produce more variability in experimental outcome, necessitating greater numbers. Studies involving NHPs are also limited to a smaller number of animals, which can negatively influence statistical power. Finally, and most importantly, any study involving NHPs is subject to ethical concerns, concerns for the wellbeing of the animals and ever-growing societal and political pressures. Any study using NHPs will require specialist facilities and trained staff, including veterinary staff.

### 1.2 Marmosets in biomedical research

Marmosets have been used in biomedical research for many decades. Over the past twenty or so years, marmoset research has increased in pace with biomedical research in general, driven in part by a growing inventory of reagents and analytical tools. Notable advances include the sequencing of the marmoset genome (Worley et al., 2014), the generation of transgenic animals by germline transmission (Sasaki et al., 2009; Tomioka et al., 2017a; Tomioka et al., 2017b), the creation of gene knockout marmoset models (Kumita et al., 2019; Yoshimatsu et al., 2019) and an ever-growing array of marmoset-specific reagents, including microarrays (Datson et al., 2007), ELISA and ELISPOT assays (Zhu et al., 2016), and monoclonal antibodies (Kametani et al., 2009). A number of marmoset-specific monoclonal antibodies are available commercially; however, these are specific to a few targets and conjugated to only few commonly used fluorophores. In spite of the challenges presented by reagent availability and technical issues, the marmoset has been utilized as an appropriate animal model in a number of contexts, including infectious disease, autoimmunity, neurobiology and, more traditionally, in developmental biology, reproductive biology, toxicology/drug development, and behavioral research. Since the focus of this review is infectious disease, a comprehensive discussion of each of these areas of research is simply not feasible. The reader is directed to a number of excellent review articles, which outline the value of the marmoset in these contexts (Mansfield, 2003; ‘T Hart et al., 2012; Okano et al., 2012; Han et al., 2022; Inoue et al., 2022).

Marmoset models utilized in neuroscience, behavioral science and reproductive biology are very well characterized, and there is a wealth of published literature in these areas. In contrast, one area that remains relatively unexplored is the marmoset immune system and the mechanisms of immune regulation. As noted, this is partly due to the limited availability of analytical tools and reagents that cross-react with the marmoset. Given their phylogenetic similarity to humans, the marmoset immune system is likely more similar to our own than that of a mouse. Nevertheless, much of our understanding of the molecular basis of the human immune system has been elucidated or predicted using murine experimental models. Thus, to understand the value of the marmoset in immunology research, a more in-depth characterization of the marmoset immune system is required. Such an endeavor would lead to the development of a wider array of analytical tools and reagents specific for the marmoset. A greater characterization of the marmoset immune system would benefit a number of existent marmoset disease models. In the proceeding section, important immunological features that are relevant to the study of infectious disease are outlined, with an emphasis on the reagents and techniques developed for the marmoset.

## 2 Marmoset immunology: like mice and man?

To best utilize the marmoset in immunological research, we need to understand the marmoset immune system. To use the marmoset as a surrogate of human diseases and conditions, we need to be confident that what we see in the marmoset actually recapitulates what we see in humans. Though many aspects of marmoset immunology remain elusive, several important findings that highlight the similarities and differences between marmoset and man have been reported over the years.

The ability of the immune system to recognize foreign (non-self) antigens is central to the adaptive immune response. One indicator of an immune systems breadth is the variability of the molecules involved in antigen recognition, [i.e., major histocompatibility complex (MHC) molecules, T-cell receptors (TCRs) and immunoglobulins (Igs)] (Kametani et al., 2018). The structure of the MHC in the marmoset has been elucidated. In the marmoset, class I MHC molecules are encoded by Caja genes (*Caja-B*, *Caja-G*, *Caja-F* loci), which are orthologs of the human leukocyte antigen (HLA) genes (classical: *HLA-A*, *HLA-B*, *HLA-C*; non-classical: *HLA-G*, *HLA-E*) in humans (Shiina et al., 2011). *Caja* genes exhibit a high degree of homology with human *HLA* genes, particularly *Caja-G* and *HLA-G*, which are evolutionarily closely related (Kametani et al., 2018). Importantly, *HLA* orthologs have not been identified in rodents (Kametani et al., 2018), reflecting the increased evolutionary distance between mouse and man. In spite of these similarities, marmoset *Caja* genes are associated with multiple alleles at each locus, but the diversity is nevertheless limited in the marmoset compared to that in man (Shiina et al., 2011; Kono et al., 2014). Furthermore, the human homolog of *Caja-G* (i.e., *HLA-G*) is a non-classical MHC molecule, represented by a single gene locus with a low number of alleles. The expression of *Caja-G* is restricted to cells of the placenta and on certain regulatory T-cells (Ferreira et al., 2017; Kametani et al., 2018; Zhuang et al., 2021). *HLA-G* has been suggested to possess immunosuppressive functions (Lin and Yan, 2016). Conversely, in the marmoset, *Caja-G* is ubiquitously expressed and polymorphic, more akin to human classical class I HLA molecules (Van Der Wiel et al., 2013; Kono et al., 2014; Li et al., 2014a; Neehus et al., 2016). The specific function of *Caja-G* in the marmoset in unclear, but it may possess immune activating functions (Münz et al., 1999; Neehus et al., 2016; Kametani et al., 2018). Uncovering the role of *Caja-G* in the marmoset may provide valuable insight into the immunological mechanisms in the species. Orthologs of the genes encoding *HLA-G* ligands in man (*LILRB1* and *LILRB2*) have also been predicted (Kametani et al., 2018). Aside from the class I HLA molecules, functional homologs of human *HLA-DR* and *HLA-DQ* (both encoding class II MHC molecules) are present in the marmoset but, relative to humans, the diversity of these molecules is restricted (Antunes et al., 1998). Nevertheless, the function of these class II orthologs appears to be similar to their human counterpart (Kametani et al., 2018). Evidence to support the divergence of *Caja-DRB* and the DRB*W16 allele in the marmoset has been reported (Prasad et al., 2006; Prasad et al., 2007). Aside from HLA molecules, the homology of the TCR repertoire between humans and marmosets is high, displaying a greater than 90% homology between man and marmoset in the *CDR3-FR4* region (Matsutani et al., 2011; Kitaura et al., 2012). Homology of human and marmoset immunoglobulins are yet to be fully-characterized. Yet, in a recent study of primate genomes and transcriptomes by Olivieri and colleagues, immunoglobulin genes were identified (Olivieri and Gambón Deza, 2018). In the marmoset, an isotype of each class of immunoglobulin was identified. Notably, the CH_2_ exon of the IgD gene is absent in the marmoset, whilst the CH_1_ and CH_3_ exons are evolutionarily conserved (Olivieri and Gambón Deza, 2018). The diversity of the B-cell response in the marmoset is, however, predicted to be more restricted (Griffiths et al., 2006; Kametani et al., 2018). For those molecules involved in immune effector responses (e.g., cytokines), complementary DNA (cDNA) sequences and amino acid sequences between marmosets and humans were 86% identical, compared with 61% between mouse and humans (Kohu et al., 2008). Numerous approaches have been adopted for the analysis of marmoset cytokines and chemokines, measuring the level of expression at the protein (i.e., by enzyme-linked immunosorbent assays (ELISAs) and cytometric bead arrays (CBAs)) and mRNA (i.e., by quantitative polymerase chain reaction (qPCR)) level (Fujii et al., 2013; Jagessar et al., 2013; Ngugi et al., 2022). The assessment of intracellular cytokines has also been performed using flow cytometric techniques (Mietsch et al., 2020). A list of assays designed for analysis of serum cytokines and chemokines that are reported to work in the marmoset are presented in **Table 2**.

**Table 2:** ELISA and CBA kits for analysis of serum cytokines and chemokines in the common marmoset.

Cytokine/Chemokine	Provider	Reference
IL-1β	BD Biosciences	(Ireland et al., 2022)
IL-2	U-CyTech, Invitrogen	(Jagessar et al., 2013; Peters et al., 2023)
IL-4	Invitrogen	(Peters et al., 2023)
IL-6	U-CyTech, BD Biosciences	(Nelson and Loveday, 2014; Ireland et al., 2022)
IL-8	Invitrogen	(Peters et al., 2023)
IL-10	U-CyTech	(Jagessar et al., 2013)
IL-13	U-CyTech	(Jagessar et al., 2013)
IL-12/23p40	U-CyTech, Pharmingen, Invitrogen	(Laman et al., 1998; Jagessar et al., 2013; Peters et al., 2023)
IL-17A	U-CyTech	(Jagessar et al., 2013; Jagessar et al., 2015; Kap et al., 2015)
IFN-γ	U-CyTech, Mabtech, Invitrogen	(Jagessar et al., 2013; Jagessar et al., 2015; Ireland et al., 2022; Peters et al., 2023)
TNF-α	U-CyTech, Mabtech, Invitrogen	(Seehase et al., 2012; Jagessar et al., 2013; Nelson and Loveday, 2014; Jagessar et al., 2015; Ireland et al., 2022; Ngugi et al., 2022; Peters et al., 2023)
MIP-1α	BD Biosciences	(Nelson and Loveday, 2014)
MIP-1β	BD Biosciences, Invitrogen	(Seehase et al., 2012; Nelson and Loveday, 2014; Peters et al., 2023)
MCP-1	BD Biosciences, Invitrogen	(Nelson and Loveday, 2014; Ireland et al., 2022; Peters et al., 2023)
RANTES	BD Biosciences	(Ireland et al., 2022)
ICAM	Invitrogen	(Peters et al., 2023)
GM-CSF	Invitrogen	(Peters et al., 2023)

CBA, cytometric bead array; CM, common marmoset; ELISA, enzyme-linked immunosorbent assay; GM-CSF, granulocyte-macrophage colony stimulating factor; ICAM, intracellular adhesion molecule; IFN, interferon; IL, interleukin; MCP, monocyte chemoattractant protein; MIP, macrophage inflammatory protein; RANTES, regulated upon activation, normal T cell expressed and presumably secreted.

To understand the process of immune cell differentiation in the marmoset, there is a need to understand the primate hematopoietic system, and how this compares to humans. The markers CD34 and CD117 are used to identify hematopoietic stem cells (HSCs) in mice and humans (Okada et al., 1992; Galy et al., 1995). Human HSCs are CD34+ CD117lo, whereas mice HSCs are CD34- CD117+ (Papayannopoulou et al., 1991; Okada et al., 1992; Gunji et al., 1993; Galy et al., 1995; Mestas and Hughes, 2004). Identification and characterization of marmoset HSCs was made possible by the development of anti-marmoset CD34 and CD117 monoclonal antibodies (Izawa et al., 2004; Kametani et al., 2009; Shimada et al., 2015). Marmosets are reported to express both CD34 and CD117; however, the differentiation of CD117+ cells into cells of the erythroid and myeloid (but not lymphoid) lineages was not dependent on CD34 expression (Ito et al., 2002; Matsumura et al., 2003; Kametani et al., 2006; Ito et al., 2008a). Whilst the specific biological function of CD34 is unclear in humans, in the marmoset it may enhance engraftment following HSC transplantation, like the situation in humans (Kametani et al., 2018). When human HSCs were transplanted into NOG immunodeficient mice, B-cell development preceded T-cell development and CD4 and CD8 T-cells developed simultaneously (Ito et al., 2002; Yahata et al., 2002; Matsumura et al., 2003; Kametani et al., 2006). In contrast, following transplantation of marmoset HSCs into NOG mice, CD8 T-cell development occurred predominantly, with no B-cell or CD4 T-cell development (Kametani et al., 2018). These findings illustrate a key species difference in the hematopoietic system between human and marmoset. Efforts should be taken to understand how this difference might influence the function of the immune system.

A significant hurdle in the study of marmoset immunology is the lack of specific reagents and analytical tools. The limited availability of marmoset-specific monoclonal antibodies is particularly problematic and limits our ability to survey the immunological landscape of the marmoset. Unsurprisingly, increased interest in the marmoset in biomedicine has led to a number of groups developing and evaluating reagents (including monoclonal antibodies) designed specifically for the marmoset, leading to the commercial availability of marmoset reagents. Nevertheless, whilst progress has been made, there remains a pressing (and as of yet unmet) need for the wider availability of validated anti-marmoset antibodies. A comprehensive discussion of these reagents is beyond the scope of this review. However, a number of marmoset-specific antibodies against common surface antigens are reported in the literature, including anti-marmoset CD45, CD3, CD4, CD8 and CD25 (Brok et al., 2001; Ito et al., 2008b; Kametani et al., 2009; Jagessar et al., 2013; Neumann et al., 2016; Gordeychuk et al., 2018). Marmoset-specific anti-CD34 and anti-CD117 antibodies were also developed as described earlier (Izawa et al., 2004; Kametani et al., 2009; Shimada et al., 2015). Whilst this list is by no means exhaustive, it is worth pointing out that, to the best of our knowledge, the only marmoset-specific monoclonal antibodies currently available commercially recognize and bind CD45 and CD8. Unfortunately, the availability of fluorochromes for conjugation is limited. Numerous studies have evaluated anti-human monoclonal antibodies for cross-reactivity with marmoset antigens. Indeed, one report showed that 126 out of 331 monoclonal antibodies tested cross-reacted with peripheral blood mononuclear cells (PBMCs) from the marmoset (Brok et al., 2001). More recently, Neumann and colleagues evaluated a panel of 120 monoclonal antibodies for cross-reactivity against the marmoset, including testing of 97 different antibody clones (49 of which were not tested previously) against cell-surface markers, intracellular markers, chemokine receptors and cytokines (Neumann et al., 2016). Finally, it should be noted here that, despite the similarities between the human and the marmoset in terms of immune molecules, not all anti-human antibodies will cross-react with the marmoset; likewise, anti-marmoset CD4 and CD8 antibodies failed to cross-react with the corresponding antigen in humans (Gordeychuk et al., 2018). It is pivotal that care is taken to properly design, test and validate immunophenotyping panels, giving researchers the assurance and confidence in the data they generate.

An in-depth, comprehensive picture of the marmoset immune system is still lacking, though a snapshot of fundamental cellular immune components in healthy animals has begun to emerge (Nelson and Loveday, 2014; Neumann et al., 2016; Gordeychuk et al., 2018; Mietsch et al., 2020; Ngugi et al., 2022). All data discussed here relate to marmoset whole blood since data from other tissues is limited. Briefly, the constitution of the marmoset immune system is remarkably similar to our own: in blood, the majority (over 80%) of cells express CD45, the common leukocyte antigen; monocytes represent a minor proportion of CD45+ cells (<5%), whilst over 40% of cells were lymphocytes (Ross et al., 2012; Nelson and Loveday, 2014; Neumann et al., 2016; Gordeychuk et al., 2018; Mietsch et al., 2020; Ngugi et al., 2022). In terms of the distribution of immune cell subsets, reports from numerous research groups, including two from our own, are largely agreeable: total T-cells (CD3+) represent between 50 and 70% of lymphocytes, with between 20 and 30% of cells being B-cells (CD20+); the frequency of natural killer (NK) cells and γδ T-cells is low (<5%); within the CD3 T-cell compartment, 50 to 60% and 30 to 40% of cells express either the CD4 or CD8 co-receptors, respectively; and a small proportion of cells (<3%) express both CD4 and CD8 (Nelson and Loveday, 2014; Neumann et al., 2016; Gordeychuk et al., 2018; Mietsch et al., 2020; Ngugi et al., 2022). In one report, the frequency of cytotoxic T-cells (CD8+) was reported to be significantly higher in the marmoset than that seen in humans (Fujii et al., 2013), possibly due to the small number of animals and/or the CD8 antibody clone. Finally, neutrophils comprised approximately 35% of circulating cells (Nelson and Loveday, 2014; Neumann et al., 2016; Gordeychuk et al., 2018; Mietsch et al., 2020; Ngugi et al., 2022). Taken together, the frequency of immune cells in the marmoset mirrors humans better than how mice mirror humans.

## 3 Modelling infectious diseases in the marmoset: tularemia, melioidosis and hepatitis C virus

The marmoset has been used as an experimental model of several infectious diseases; this information, along with a summary of both the number of studies utilizing a marmoset disease model and alternative animal models, is provided in **Table 3**. A comprehensive discussion of each of these models is beyond the scope of this manuscript, thus the final section of this review will examine two experimental models of bacterial infection and one of viral infection that have been successfully developed in the marmoset: *Francisella tularensis* and *Burkholderia pseudomallei*, the etiological agents of tularemia and melioidosis, respectively, and hepatitis C virus (HCV) and the related GB virus B (GBV-B). Tularemia and melioidosis (and their respective causative agents) were selected for discussion given their potential for use as biological warfare agents; hepatitis C was selected since the marmoset has been shown to be susceptible to infection and therefore represents an important surrogate model. Whilst a discussion of the marmoset models of Ebola, Zika and influenza viruses would have been extremely interesting, these agents were not selected for further discussion in this review.

**Table 3:** Marmoset models of infectious disease.

Infectious Agent/Disease	Studies reporting marmoset model	Alternative animal models	References
Lassa	Three studies (model development and characterization and vaccine efficacy)	Mouse, Squirrel monkey, Cynomolgus macaque, Rhesus macaque, Guinea pig	(Carrion et al., 2007; Lukashevich et al., 2008; Zapata et al., 2014; Sattler et al., 2020)
Hepatitis C virus (type species within the genus *Hepacivirus*) and the closely related species GB virus B	Many studies GB virus B infects small New World primates only; marmoset model is a surrogate model for human HCV	Chimpanzee, Tree shrew, Mouse	(Bukh et al., 2001; Lanford et al., 2003; Bright et al., 2004; Guha et al., 2005; Kyuregyan et al., 2005; Brass et al., 2007; Haqshenas et al., 2007; Weatherford et al., 2009)
Dengue virus	Many studies (model development and characterization and vaccine efficacy)	Mouse, Swine, Rhesus macaque, Chimpanzee, Tree Shrew	(Omatsu et al., 2011; Omatsu et al., 2012; Yoshida et al., 2013; Moi et al., 2014; Moi et al., 2017; Na et al., 2017; Muhammad Azami et al., 2020; Jiang et al., 2021)
Herpesviruses	One study (model characterization)	Mouse, Pig-tailed macaque	(Lusso et al., 1994; Lusso et al., 2007; Leibovitch et al., 2013; Horvat et al., 2014; Reynaud et al., 2014)
Junin virus (Argentine hemorrhagic fever)	Many historical publications from 1980s (model development and characterization and vaccine efficacy)	Guinea pigs	(Weissenbacher et al., 1979; Weissenbacher et al., 1982; González et al., 1983; Molinas et al., 1983; Avila et al., 1985; Weissenbacher et al., 1986; Avila et al., 1987)
Rift valley fever	Four studies (model development and characterization and vaccine efficacy)	Rodents, Sheep, Goats, Cattle, Rhesus macaque	(Peters et al., 1988; Morrill et al., 1990; Smith et al., 2012; Hartman et al., 2014; Smith et al., 2018; Wichgers Schreur et al., 2022)
Severe acute respiratory syndrome (SARS) (including SARS-coronavirus (CoV)2 (COVID-19))	Many studies (model development, characterization and vaccine/therapeutic efficacy)	Mouse, Golden hamster, Ferret, Rhesus monkey, African green monkey, Baboon, Pig	(Greenough et al., 2005; Lu et al., 2020; Albrecht et al., 2021; Renn et al., 2021; Singh et al., 2021; Trichel, 2021; Da Costa et al., 2022; Fan et al., 2022; Ireland et al., 2022; Lin et al., 2022)
Middle East respiratory syndrome (MERS)	Many studies (model development, characterization and vaccine/therapeutic efficacy)	Mice, Syrian hamsters, Ferrets, Rabbits, Rhesus monkey	(Raj et al., 2013; Falzarano et al., 2014; Chan et al., 2015; Johnson et al., 2015; Van Doremalen and Munster, 2015; Chen et al., 2017; Van Doremalen et al., 2017; Yu et al., 2017; De Wit et al., 2018; Nelson et al., 2022b)
Eastern equine encephalitis virus (EEEV)	Two studies (model development and characterization)	Mouse, Hamsters, Cynomolgus macaque	(Jackson et al., 1991; Adams et al., 2008; Steele and Twenhafel, 2010; Porter et al., 2017; Phelps et al., 2019; Burke et al., 2022)
*Bacillus anthracis* (anthrax)	Two studies (model development and characterization and therapeutic efficacy)	Mouse, Guinea pigs, Rabbits, Cynomolgus monkey	(Lever et al., 2008; Nelson et al., 2011b; Ben-Shmuel et al., 2018; Perry et al., 2020; Stratilo et al., 2020; Gates-Hollingsworth et al., 2022)
*Francisella tularensis* (tularemia)	Three studies (model development, characterization and therapeutic/vaccine efficacy)	Humans, Mice, Rats, Rabbits, Guinea pigs, Cynomolgus monkey, Grivet monkey, Rhesus monkey	(Rick Lyons and Wu, 2007; Nelson et al., 2009; Nelson et al., 2010a; Nelson et al., 2010b)
*Burkholderia pseudomallei* (melioidosis) and *Burkholderia mallei* (glanders)	Eight studies (model development, characterization and therapeutic efficacy)	Mouse, Goats, African green monkey, Rhesus monkey, Invertebrates	(Woods, 2002; Nelson et al., 2011a; Rowland et al., 2012a; Soffler et al., 2012; Laws et al., 2013; Nelson et al., 2014; Nelson et al., 2015; Ganesan et al., 2020; Nelson et al., 2021; Trevino et al., 2021; Nelson et al., 2022a; Ngugi et al., 2022)
Marburg virus	Two studies (model development and characterization)	Cynomolgus monkey, Rhesus monkey, Mouse, Hamster, Guinea pig	(Carrion et al., 2011; Smither et al., 2013; Glaze et al., 2015; Shifflett and Marzi, 2019)
Ebola virus	Two studies (model development and characterization)	Mouse, Hamsters, Guinea pigs, Ferrets, Macaque monkey, African green monkey, Baboon	(Carrion et al., 2011; Nakayama and Saijo, 2013; Willyard, 2014; Shurtleff and Bavari, 2015; Smither et al., 2015; St Claire et al., 2017; Longet et al., 2020)
Orthopoxviruses, e.g., variola virus (smallpox) and monkeypox virus	Five studies (model development and characterization)	Mouse, Rabbit, Cynomolgus monkey, African dormouse, Ground squirrel	(Smee, 2008; Kramski et al., 2010; Goff et al., 2011; Mätz-Rensing et al., 2012; Mucker et al., 2015; Schmitt et al., 2017; Mucker et al., 2018)
*Coxiella burnetii* (Q fever)	One study (model development and characterization)	Mouse, Guinea pigs, Cynomolgus monkey, Rhesus monkey	(Bewley, 2013; Gregory et al., 2019; Nelson et al., 2020)
Zika virus	Six studies (model development, characterization and vaccine efficacy)	Mouse, Rhesus monkey, Cynomolgus monkey	(Bradley and Nagamine, 2017; Chiu et al., 2017; Kublin and Whitney, 2018; Lum et al., 2018; Seferovic et al., 2018; Terzian et al., 2018; Berry et al., 2019; Luo et al., 2020; Kim et al., 2022)
West Nile virus	One study (model development and characterization)	Mouse, Baboon, Goose, America singer canaries, Rabbits, Zebra finch	(Wolf et al., 2006; Bowen and Nemeth, 2007; M, S. E. S et al., 2013; Verstrepen et al., 2014; Suen et al., 2015; Graham et al., 2017; Hofmeister et al., 2017; Hofmeister et al., 2018)
Bovine spongiform encephalopathy (BSE)	Five historical publications details (model development and characterization)	Sheep	(Done, 1992; Morris, 1992; Whitaker, 1992; Baker et al., 1993; Bradley, 1993; Hunter, 2003)

### 3.1 Animal models of infectious disease: introducing the 3 R’s and the animal efficacy rule

For many infectious diseases, disease incidence is too low to model in human populations. Studies involving humans are obviously subject to significant ethical concerns and, where diseases are fatal, human challenge studies are impossible. Nevertheless, modelling the efficacy of a potential medical countermeasure is a crucial step towards drug/therapy licensure (Gronvall et al., 2007; Dicarlo et al., 2011; Aebersold, 2012). Animal models are frequently used in an attempt to better understand disease pathogenesis in humans and to support both the identification of diagnostic correlates and effective treatment regimens (Gronvall et al., 2007). The use of animals in scientific research is tightly regulated and animals are used for research within an ethical framework. In the United Kingdom (UK), the Animals (Scientific Procedures) Act 1986 extends this ethical framework by imposing a set of comprehensive legal requirements for any institution wishing to undertake research involving animals (Hollands, 1986). In essence, research proposals involving animals are carefully reviewed to assess factors such as any harm animals might incur, the protocols and procedures involved, the number and types of animal used and the value of the study in terms of the potential benefits. Additionally, UK government introduced additional controls in 1998, namely the Ethical Review Process, with the aims of providing independent ethical advice for projects (Pietrzykowski, 2021). This move to promote an ethical analysis of a project and to enhance awareness of animal welfare issues is a fundamental part of engaging with the concept of the 3R’s (*replacement*, *reduction* and *refinement*) (Russell and Burch, 1959; Fenwick et al., 2009; Hubrecht and Carter, 2019). In a recent monography, ‘t Hart proposed a fourth R: *relevance* and particularly the clinical relevance of an animal model (‘T Hart, 2019). It is perhaps the relevance where the marmoset excels over murine models of infection. The FDA established the animal efficacy rule (or simply the animal rule) in 2002; this was later authorized by the United States Congress (Allio, 2018). The animal efficacy rule applies to all studies that aim to develop and/or test the efficacy of a given therapy against a life-threatening or life-changing biological, chemical, radiological or nuclear agent and where human efficacy trials are either unethical or not feasible.

### 3.2 Francisella tularensis

*Francisella tularensis* is a small, gram-negative, facultative intracellular coccobacillus and the causative agent of tularemia in humans (Wayne Conlan and Oyston, 2007). The bacterium was first isolated in 1911 from ground squirrels in Tulare County, California, and later in 1914 from a human in Ohio (Mccoy and Chapin, 1912; Wherry and Lamb, 1914). Three subspecies have been described: i) subsp. *tularensis* (type A strains), ii) subsp. *holarctica* (type B strains), and iii) subsp. *mediasiatica*; a fourth strain, generally considered a separate species given its aquatic reservoir and low virulence in humans, is *F. novicida* (Caspar and Maurin, 2017). Type A and B strains are responsible for the vast majority of tularemia cases in humans, with the type A strain being most virulent (Maurin, 2015). *F. tularensis* is a highly pathogenic organism that can cause severe and sometimes fatal disease in humans. An important aspect to *F. tularensis* virulence is its ability to replicate within eukaryotic cells, such as in the cytosol of macrophages (Steiner et al., 2014). Tularemia is a zoonotic disease; cases of the disease are typically sporadic or occur in small familial groups (Tärnvik and Berglund, 2003; Janse et al., 2018). Infection occurs via direct contact with infected animals, consumption of contaminated food or water, exposure to contaminated environments or via arthropod bites (e.g., mosquitoes and tics) (Keim et al., 2007; Carvalho et al., 2014). Lagomorphs and small rodents are the primary hosts of the pathogen (Maurin and Gyuranecz, 2016).

Tularemia symptoms vary depending on the route of exposure; six clinical forms of the disease have been described, namely: i) ulceroglandular, ii) glandular, iii) oropharyngeal, iv) oculoglandular, v) pneumonic, and vi) typhoidal (Yeni et al., 2021). Ulceroglandular and glandular forms (with or without skin ulcers at the inoculation site, respectively) result from skin exposure (e.g., via arthropods) and patients present with regional lymphadenopathy (Caspar and Maurin, 2017; Balestra et al., 2018). Oculoglandular tularemia results from exposure via the ocular conjunctiva and patients typically present with painful conjunctivitis and regional lymphadenopathy (Kantardjiev et al., 2007). Oropharyngeal tularemia usually results from ingestion of contaminated meat or water, leading to pharyngitis and regional lymphadenopathy (Steinrücken and Graber, 2014). Patients presenting with the pneumonic form of disease, caused by inhalation of airborne particles, experience cough, fever and dyspnea; mediastinal or hilar lymphadenopathy is sometimes observed (Gill and Cunha, 1997; Williams et al., 2019). Finally, typhoidal disease is characterized by systemic disease with neurological manifestations that mimic the symptoms of typhoid. Frequently, no symptoms of localized infection are observed, nor is the site of bacterial entry (Faucher et al., 2012). Complications of infection with *F. tularensis* include skin eruptions, abscess formation, suppuration of lymph nodes and the emergence of secondary infectious locations.

The potential of airborne transmission of *F. tularensis* infection, its ability to cause severe human disease and low infectious dose has led to the bacterium’s classification as a potential bioterrorism agent (Dennis et al., 2001). Diagnosis is challenging and is based on clinical and epidemiological features, serological tests and detection of microbial DNA by PCR. Since the isolation of the bacterium from blood and tissues of infected individuals occurs in less than 20% of cases, antibiotic susceptibility testing is difficult (Maurin et al., 2011). Treatment of tularemia is with antibiotics; the aminoglycosides, fluoroquinolones or tetracycline classes of antibiotic are recommended (Dennis et al., 2001; Ellis et al., 2002). No licensed tularemia vaccine is currently available, although a live attenuated vaccine is still in use in certain parts of the world where it is reserved to treat the most at-risk persons.

#### 3.2.1 Common marmoset model of tularemia

A number of animal models of *F. tularensis* infection have been developed, including mice, rats, rabbits, guinea pigs and non-human primates (e.g., cynomolgus and rhesus monkeys). The advantages and disadvantages of these various animal models (and how they compare with the marmoset model) are presented in **Table 4**. To the best of our knowledge, we are the only group to report on a marmoset model of *F. tularensis* infection to-date (Nelson et al., 2009; Nelson et al., 2010a; Nelson et al., 2010b). In this section, the marmoset model of inhalational tularemia will be discussed with a particular emphasis on the immunological features. The reader is directed to the above publications for full details of the model.

**Table 4:** Marmoset and alternative models of *Francisella tularensis* infection (tularemia).

Marmoset model of *Francisella tularensis* infection			
Advantages	Disadvantages	Reference	
Similar disease course and pattern of organ involvement to human disease and to disease in other non-human primates Natural susceptibility of captive marmosets to infection Low infectious dose Highly susceptible to infection by airborne route Reproducibility	Limited numbers of animals per study More compressed disease course compared to humans Need for more studies utilizing marmoset model of infection – with particular emphasis on the host immune response and how this compares to humans Lack of studies assessing efficacy of therapeutics and candidate vaccines	(Posthaus et al., 1998; Splettstoesser et al., 2007; Nelson et al., 2009; Nelson et al., 2010a; Nelson et al., 2010b; Antwerpen et al., 2013)	

Alternative animal models of *Francisella tularensis* infection			
Model	Advantages	Disadvantages	Reference
Humans	Safe to perform in several hundred volunteers Low dose of pathogen to induce infection Reproducible incubation period and clinical course Translatable model for assessment of antibiotic and vaccine efficacy	Public perceptions of human trials, particularly with biowarfare agents Ethical concerns of using humans; such studies not possible today	(Stuart and Pullen, 1945; Rick Lyons and Wu, 2007; Hepburn and Simpson, 2008; Oyston and Griffiths, 2009)
Non-human primates	Best recapitulates human disease, particularly in terms of LVS-induced protection against type A strains and the development of skin ulcers Pattern of organ involvement similar to that in humans Infection with certain type B strains often self-limiting as in humans	More technically challenging and expensive Enhanced sensitivity and limited resistance to type B strains compared to humans	(Sawyer et al., 1966; Day and Berendt, 1972; Baskerville et al., 1978; Hambleton et al., 1978; Rick Lyons and Wu, 2007; Twenhafel et al., 2009; Stundick et al., 2013; Roberts et al., 2018)
Mice	Cheap and readily available Well-characterized genetics Genetically manipulated (e.g., gene knock-out) mice available Wide availability of immunological reagents and tools Protection afforded by RML LVS vaccine strain	Conflicting reports concerning how mouse pathology relates to human disease Sensitive to LVS LVS-induced protection is temporary; little-to-no protection afforded by LVS against SCHU S4 strain	(Twine et al., 2006; Rick Lyons and Wu, 2007; Conlan et al., 2008; Conlan et al., 2010; Rozak et al., 2010; Shen et al., 2010; Twine et al., 2012)
Rats	Intradermal and aerogenic inoculation with LVS confers protection Low infectious dose Similar pathology and organ involvement	Limited number of studies Animals susceptible to infection but typically recover Natural resistance to LVS and SCHU S4 strains	(Dennis et al., 2001; Lamps et al., 2004; Rick Lyons and Wu, 2007; Wu et al., 2009; Ray et al., 2010; Signarovitz et al., 2012; Chu et al., 2014; Hutt et al., 2017)
Rabbits	Natural host of bacterium Similar susceptibility to humans Pathology recapitulates human disease Resistance to type B strains	Limited number of studies and data although increasing Limited availability of immunological reagents and tools Conflicting reports of LVS vaccine efficacy	(Baskerville and Hambleton, 1976; Rick Lyons and Wu, 2007; Pasetti et al., 2008; Reed et al., 2011; Reed et al., 2014; Brown et al., 2015; Stinson et al., 2016)
Guinea pigs	Sensitive to SCHU S4 (type A) strain	Limited number of studies and data Limited model characterization Conflicting reports of LVS vaccine efficacy Limited availability of immunological reagents and tools	(Eigelsbach and Downs, 1961; Eigelsbach et al., 1961; Rick Lyons and Wu, 2007)

LVS, Live vaccine strain.

The marmoset as an NHP model of tularemia has a number of advantages (see **Table 4**); importantly, the course and progression of disease accurately recapitulated human disease – including the development of ulcers, a feature not observed in any other animal model (Nelson et al., 2010b; Roberts et al., 2018). Evidence of an immune response was demonstrated by the production of pro-inflammatory cytokines with disease progression. For example, at 72 hrs post-challenge, monocyte chemoattractant protein (MCP)-1 (CCL2) was detectable in the spleen, lungs and blood and the level increased until death (Nelson et al., 2010b). Additional cytokines, including macrophage inflammatory protein (MIP-1α; CCL3), MIP-1β (CCL4), interleukin (IL-6), IL-1β and regulated on activation, normal T-cell expressed and secreted (RANTES; CCL5), were upregulated in all organs at 96 hrs post-challenge (Nelson et al., 2010b). Interestingly, MIP-1α and IL-6 were first observed shortly prior to death, akin to the murine model of inhalational tularemia (Conlan et al., 2008; Nelson et al., 2010b). Neutrophils and natural killer (NK) cells were the first cells to arrive at the site of infection (24 hrs post-challenge), followed by macrophages, T-cells and additional influx of NK cells (48 hrs post-challenge) (Nelson et al., 2010b). A decline in the percentage of neutrophils in the lung and blood at 72 hrs post-challenge was observed, raising important questions concerning the role of neutrophils in response to *F. tularensis* infection. Indeed, studies assessing the importance of neutrophils in the response to *F. tularensis* infection are conflicting. Using the neutrophil-depleting antibody RB6-8C5, Sjöstedt and colleagues found that mice depleted of neutrophils were vulnerable to otherwise sublethal doses of *F. tularensis*, delivered either intraveneously or intradermally, suggesting a key role for neutrophils in controlling bacterial replication (Sjöstedt et al., 1994). Meanwhile, KuoLee and colleagues demonstrated that depleting the number of neutrophils had no effect on the bacterial burden or time to death (Kuolee et al., 2011). It has been suggested that the role of neutrophils in response to infection with *F. tularensis* may be dependent on the site of infection and that, in some cases, excessive neutrophil recruitment may contribute to the over-production of pro-inflammatory cytokines that ultimately lead to sepsis (Malik et al., 2007; Metzger et al., 2013; Steiner et al., 2014). Notably, whilst infection with the type A strain rapidly induced neutrophil recruitment in the marmoset, the type B (but not type A) strain led to neutrophil influx in the mouse, highlighting an important difference between the two species (Hall et al., 2008; Nelson et al., 2010b). By 72 hrs post-challenge, the number of B-cells and T-cells in the spleen and blood increased (Nelson et al., 2010b). By 96 hrs post-challenge, the number of neutrophils in the blood and organs returned to normal levels; a concomitant decline in the number of NK cells, both B- and (CD4+) T-lymphocytes and macrophages in the lungs was also observed (Nelson et al., 2010b). The proportion of CD8+ T-cells and γδ T-cells in the spleen and lung were increased 96 hrs post-challenge (Sumida et al., 1992; Poquet et al., 1998; Kroca et al., 2000; Nelson et al., 2010b). γδ T-cells are thought to play a role in the innate immune response and thought to be important in human infections with *F. tularensis* (Rowland et al., 2012a; Rowland et al., 2012b). An increase of γδ T-cells in the blood was not observed, consistent with reports in humans, where cells were discerned approximately one week post-infection (Kroca et al., 2000).

Having shown the marmoset model of tularemia effectively recapitulates human disease, a follow-up study by our research group evaluated the efficacy of levofloxacin, a fluoroquinolone shown to be effective against *F. tularensis* (Hepburn and Simpson, 2008). Fluoroquinolones have a number of advantages over current treatment protocols, including their broad-spectrum activity (important when diagnosis is difficult), bactericidal effects, tolerability and oral administration (Fish, 2003; Hepburn and Simpson, 2008). Further, levofloxacin is effective as a single daily dose which will likely increase compliance (Nelson et al., 2010a). Indeed, levofloxacin is approved for the treatment of inhalational anthrax in both children and adults (Deziel et al., 2005; Li et al., 2010). To achieve licensure of any therapeutic agent for a given disease under the animal rule (discussed earlier), the efficacy and safety profile must first be assessed in a NHP. In our study, all animals that received levofloxacin for ten days post-exposure survived and showed no clinical signs of disease, indicating the efficacy of oral levofloxacin against inhalational tularemia (Nelson et al., 2010a).

In summary, the common marmoset model of tularemia effectively and accurately recapitulates human disease and has numerous advantages over alternative animal models. It will be useful for the evaluation and licensure of medical countermeasures by the FDA.

### 3.3 Burkholderia pseudomallei

*Burkholderia pseudomallei* is a gram-negative, intracellular pathogens and the agent responsible for melioidosis (Whitlock et al., 2007; Wiersinga et al., 2018). *B. pseudomallei* is classified as Tier 1 Select Agents by the Centers for Disease Control and Prevention (CDC) given its potential use in bioterrorism (Peacock et al., 2008). Melioidosis was first described as a ‘glanders-like disease’ in 1913 by Alfred Whitmore (Whitmore, 1913). As an environmental saprophyte, *B. pseudomallei* is found in wet soils and contaminated water in endemic areas; *B. pseudomallei* is endemic in northern Australia and north east Thailand, and an emerging disease in India, China and potentially the United States (Ashdown and Clarke, 1992; Dance, 2000; Cheng and Currie, 2005; Limmathurotsakul et al., 2016). Most cases of infection occur through contact of broken skin with contaminated soil and water, although numerous other routes of exposure have been documented including ingestion and inhalation of bacteria (Webling, 1980; White et al., 1989; Abbink et al., 2001; Holland et al., 2002; Ralph et al., 2004; Baker et al., 2011; Limmathurotsakul and Peacock, 2011; Bzdyl et al., 2022). Melioidosis presents as a systemic disease; symptoms are frequently non-specific, vary from person-to-person, and can mimic several other clinical scenarios making diagnosis challenging (Yee et al., 1988; White, 2003; Cheng and Currie, 2005). The immunocompromised are particularly vulnerable to infection; risk factors for more severe disease include diabetes and lung and kidney disease (Ip et al., 1995; Northfield et al., 2002; Kronsteiner et al., 2019; Bzdyl et al., 2022). Treatment paradigms are complex and slow: an initial intensive phase requiring intravenous antibiotics (ceftazidime or meropenem) for 14 days is followed by an eradication phase, where antimicrobials (co-trimoxazole and doxycycline as combination therapy or equally efficacious co-trimoxazole monotherapy) are taken orally for a prolonged period to kill residual bacteria (Cheng et al., 2004; Chusri et al., 2012; Lipsitz et al., 2012; Chetchotisakd et al., 2014; Dance, 2014; Fisher and Harris, 2014). Disease relapse is common given the nature of the microorganism (i.e., it is intracellular and can evade the host immune response) despite prolonged antimicrobial therapy (Limmathurotsakul et al., 2008; Dance, 2014; Mariappan et al., 2021). No licensed vaccine is currently available.

#### 3.3.1 Common marmoset model of melioidosis

Despite early studies of experimental melioidosis in rhesus macaques, much of our understanding of the pathogenesis and the effectiveness of therapies against melioidosis and glanders has emerged from small animal models, specifically mice and hamsters (Warawa, 2010; Amemiya et al., 2017). Reports from the 1990s described experimental infections of baboons with *B. pseudomallei* and *B. mallei* (Manzeniuk et al., 1999). More recently, our group established and characterized a common marmoset model of *B. pseudomallei* infection following inhalational challenge (Nelson et al., 2011a). An African green monkey and rhesus macaque model of experimental infection has also been described (Miller et al., 1948; Yeager et al., 2012). The advantages and disadvantages of these various animal models, and how they compare with the marmoset model, are presented in **Table 5**. In this section, the marmoset model of melioidosis is discussed with particular emphasis on the immunological features. The reader is directed to the above publications for full details of the model.

**Table 5:** Marmoset and alternative animal models of *Burkholderia pseudomallei* infection (melioidosis).

Marmoset model of *Burkholderia pseudomallei* infection			
Advantages	Disadvantages	Reference	
Similar disease course and pattern of organ involvement to human disease Highly susceptible to infection, particularly via aerosol route Vulnerable to challenge via the subcutaneous route Severe and acute disease; animals experience fever, bacteremia and have lesions in the lung, liver and spleen Association between challenge dose and disease outcome and time to death Useful to assess efficacy of antimicrobials and vaccines Have Vγ9Vδ2 T cells, a cell type present in human melioidosis survivors	Limited reports detailing natural susceptibility of the marmoset to infection Low lethal dose and rapid time to death makes study of chronic disease impossible Primary cutaneous melioidosis in the marmoset produces severe, rapidly fatal disease (even with low doses) whereas in humans disease is rarely severe	(Warawa, 2010; Nelson et al., 2011a; Laws et al., 2013; Nelson et al., 2014; Nelson et al., 2015; Amemiya et al., 2017; Nelson et al., 2021; Ngugi et al., 2022)	

Alternative animal models of *Burkholderia pseudomallei* infection			
Model	Advantages	Disadvantages	Reference
Non-human primates	Susceptible to infection, including via the respiratory route Best recapitulate human disease, including incubation period and pattern of organ involvement	Susceptibility of infection depends on species, e.g., gorillas are highly susceptible to infection Reduced susceptibility to natural disease High cost Ethical concerns and public perception Limited availability of immunological reagents and tools	(Miller et al., 1948; Kaufmann et al., 1970; Fritz et al., 1986; Dance et al., 1992; Yap et al., 1995; Manzeniuk et al., 1999; Yeager et al., 2012; Ritter et al., 2013; Yingst et al., 2014; Amemiya et al., 2017; Waag et al., 2021)
Mice	Cheap and readily available Well-characterized genetics Genetically-manipulated mice available Wide availability of immunological reagents and tools Highly susceptible to infection via intravenous, intraperitoneal, subcutaneous and aerosol challenge Low infectious dose Similar pattern of organ involvement to humans ‘Gold-standard’ for study of disease pathogenesis and efficacy of therapies	Susceptibility to infection varies depending on mouse strain used, i.e., BALB/c mice are highly susceptible whereas C57BL/6 mice are resistant (but the latter permits study of chronic disease) Differences in physiology between mouse and humans, particularly in respiratory tract	(Dannenberg and Scott, 1958; Leakey et al., 1998; Mestas and Hughes, 2004; Tan et al., 2008; Warawa, 2010; Massey et al., 2014; Welkos et al., 2015; Amemiya et al., 2017; Bearss et al., 2017)
Hamsters	Highly susceptible to infection via intravenous, intraperitoneal, subcutaneous and aerosol challenge Identification of genetic loci associated with disease susceptibility ‘Gold-standard’ for study of disease pathogenesis and efficacy of therapies	Rapidly fatal, acute disease limits uses of model Inability to determine how route of infection impacts on disease susceptibility Reduced susceptibility to respiratory disease?	(Miller et al., 1948; Dannenberg and Scott, 1958; Ellison et al., 1969; Brett et al., 1997; Gutierrez and Warawa, 2016)
Rats	Models of septicemic and respiratory disease Streptozotocin-induced diabetes rat model is susceptible to disease Non-diabetic Sprague-Dawley rats are susceptible to respiratory infection Chronic pulmonary melioidosis model exists	Sprague-Dawley rats resistant to disease via the intraperitoneal route More resistant than mice to infection via respiratory route Somewhat limited availability of immunological reagents and tools compared to mice	(Woods et al., 1993; Van Schaik et al., 2008; Warawa, 2010)
Ferrets	Highly susceptible to infection via intravenous, intraperitoneal, subcutaneous and aerosol challenge	Lack of experimental data and well-characterized models Limited availability of immunological reagents and tools	(Miller et al., 1948)
Guinea pigs	Moderately susceptible to infection	Lack of experimental data and well-characterized models Conflicting reports of susceptibility to disease Limited availability of immunological reagents and tools	(Miller et al., 1948; Chambon, 1955; Mccormick et al., 1977; Manzeniuk et al., 1999)
Rabbits	Moderately susceptible to infection	Lack of experimental data and well-characterized models Limited availability of immunological reagents and tools	(Miller et al., 1948; Miller and Clinger, 1961)
Livestock	Natural host model Enhanced susceptibility to respiratory as opposed to systemic disease Similar to human disease	Highly resistant to natural infection; failure to establish symptomatic infection Not useful for study of chronic disease? Biocontainment concerns Tendency to develop chronic disease with granulomatous lesions	(Nicholls, 1930; Stanton and Fletcher, 1932; Cottew et al., 1952; Laws and Hall, 1963; Narita et al., 1982; Thomas et al., 1990; Vesselinova et al., 1996; Najdenski et al., 2004; Warawa, 2010; Soffler et al., 2012; Soffler et al., 2014; Amemiya et al., 2017)
Invertebrates	Likely natural disease vectors Susceptible to infection; can infect naïve guinea pigs	Limited number of studies High prevalence of *B. pseudomallei* in the environment makes it difficult to prove role of invertebrates as disease vectors	(Kharbov et al., 1981; Sulaiman et al., 2000; O’quinn et al., 2001; Schell et al., 2008; Hasselbring et al., 2011; Fisher et al., 2012; Amemiya et al., 2017)

Work from our research group has led to the development of a marmoset model of experimental melioidosis caused by three natural routes of exposure to *B. pseudomallei*, i.e., through broken skin, inhalation and ingestion (Nelson et al., 2011a; Nelson et al., 2014; Nelson et al., 2015; Nelson et al., 2021; Nelson et al., 2022a; Ngugi et al., 2022). Clinically, this is important as the route of exposure, whilst often difficult to determine at disease presentation, is likely to impact on the efficacy of medical countermeasures. Whilst early studies of experimental melioidosis in the marmoset reported limited immunological findings, a recent study by Ngugi and colleagues provided the most complete and comprehensive analysis of the immunological features of acute pneumonic disease resulting from *B. pseudomallei* exposure to-date (Ngugi et al., 2022). Significantly, features of the marmoset immune response to infection (e.g., neutrophil and macrophage migration and activation, T-cell activation and the production of pro-inflammatory mediators) mimicked acute disease in humans and was associated with disease prognosis, providing additional evidence as to the validity of the model. The proceeding section will focus predominantly on neutrophils, though other immunological components will be noted.

Notably, naïve marmoset neutrophils exhibited a rather different phenotype compared to the human counterpart. Specifically, HLA-DR (MHC II) was constitutively expressed on naïve marmoset neutrophils whereas in humans HLA-DR expression is not typically observed on resting neutrophils (Meinderts et al., 2019; Ngugi et al., 2022). Additionally, expression of the classical marker used to identify human neutrophils, CD16 (the Fc receptor gamma III), was lower on marmoset neutrophils (Silvestre-Roig et al., 2019; Ngugi et al., 2022). Considering that the proportion of circulating cells (and particularly neutrophils) in the marmoset more closely resembles that in humans, the significance of these phenotypic variations is unclear and the marmoset remains a viable model of human disease. Most importantly, both the proportions and cellular phenotypes changed during the course of the disease providing an objective, quantitative metric of disease progress and thus the opportunity to assess the efficacy of therapeutic interventions. In this study, the proportion of circulating neutrophils increased during the first 48 hrs post-challenge, after which the number declined significantly (and below baseline levels) in terminal animals. Meanwhile, the proportion of neutrophils in the lung declined 12 hrs post-challenge which is contrary to the scenario in the mouse, whereby neutrophil influx into the lung is observed post-challenge (Laws et al., 2011). At 36 hrs post-challenge, neutrophil proportion began to recover, returning to near-baseline levels by 48 hrs post-challenge. The authors noted, however, that since cell typing was proportional, it was not clear whether the apparent decline in the number of neutrophils in the lung was the result of neutrophil death [as a result of bactericidal processes (Kaplan and Radic, 2012)] or merely indicative of enhanced lymphocyte infiltration. Concomitantly, the proportion of circulating T (but not B) lymphocytes declined as the disease progressed. As noted, lymphocyte proportions were increased in the lung at 12 hrs post-challenge and continued to increase until 36 hrs post-challenge, after which levels declined. Changes to the proportions of cells in the spleen were similar to those observed in blood. In addition to changes to the proportion of cells in the various tissues, phenotypic changes were observed in neutrophils immediately following challenge. Significantly, expression of HLA-DR (which is constitutively expressed on marmoset neutrophils) dropped as disease progressed in the blood, lung and spleen. In blood, significantly reduced expression of HLA-DR was observed at all-time points post-challenge; in the lung and spleen, a significant decline in the proportion of neutrophil HLA-DR expression was observed by 12 hrs post-challenge and before the onset of clinical signs of disease, e.g., fever. Taken together, these findings provide additional evidence to support the use of the marmoset model of melioidosis for assessing medical countermeasures. Encouragingly, these findings regarding HLA-DR, CD54 and CD16 were also observed in a more recent, related study with *B. pseudomallei* (Nelson et al., 2022a).

Considering the role of neutrophils as first-responders to injury and insult, and their documented significance in early melioidosis (Easton et al., 2007; Laws et al., 2011),the fact that neutrophils showed the most significant variation of all cellular parameters assessed is not surprising. In the mouse, neutrophils play a central role in the acute response to aerosol infection (Easton et al., 2007). Though susceptibility to infection is largely pre-determined depending on the specific mouse strain (Warawa, 2010), marmosets are considered to demonstrate enhanced sensitivity to (particularly) aerosol challenge and this may be due to the tendency for a decline in the proportion of neutrophils in the lung during the early stages of infection (Nelson et al., 2022a; Ngugi et al., 2022). Alternative explanations should not be disregarded. These include the possibility that early neutrophil influx into the lung does occur, yet neutrophils are not detectable by flow cytometry because they are infected and degraded. In this scenario, subsequent neutrophil recruitment and activation occurs too late to counteract an already rapidly escalating bacterial burden. Encouragingly, the pattern of neutrophil recruitment in the marmoset mirrors that observed in other NHP models in the rhesus macaque and African green monkey (Yeager et al., 2012). Additional evidence implicating neutrophils as key players in early melioidosis include the association between excessively high or low neutrophil counts and poorer outcomes in humans, and the increased susceptibility of individuals with certain conditions (e.g., diabetes) associated with suboptimal neutrophil function (Chanchamroen et al., 2009; Saengmuang et al., 2014; Jenjaroen et al., 2015).

With a marmoset-specific candidate biomarker indicative of infection (a reduction in neutrophil HLA-DR expression), our research group recently evaluated the efficacy of co-trimoxazole using the marmoset model of experimental melioidosis (Nelson et al., 2022a). In this study, animals were challenged by one of three exposure routes: inhalational, ingestion or subcutaneous. Once fever had developed, a proportion of the animals were administered oral co-trimoxazole; all remaining animals received a placebo. A second-dose was administered 12 hrs after the first, followed by one dose every 12 hrs up until a total of 28 doses was delivered. With respect to the immunological perturbations, the proportion of neutrophils increased at the onset of fever, yet there was a drop in the level of HLA-DR expression that continued until animals succumbed to disease. HLA-DR expression was at a normal level by day 15 post-challenge in those animals that received oral co-trimoxazole. In addition to validating the observation of decreased HLA-DR expression with the onset of fever in an independent study, the immunophenotyping panel was also expanded and incorporated markers for CD16 (Fc gamma receptor III, expressed on NK cells, macrophages and neutrophils, plays a role in the internalization of exogenous antigens by binding the Fc portion of IgG immune complexes),CD66b (an activation marker on granulocytes), CD80 (a co-stimulation marker used by professional phagocytes to aid in MHC to T-cell receptor interactions) and CD54 (intracellular adhesion molecule-1 (ICAM-1), an adhesion molecule involved in lymphocyte homing and activation). Expression of all these markers decreased in the placebo group; meanwhile, neutrophil CD16 expression returned to normal levels in the co-trimoxazole treatment group. Upon treatment cessation, animals either survived, relapsed and succumbed to disease or exhibited abnormal immunological perturbations indicative of subclinical disease. Importantly, those animals that survived without relapse maintained normal levels of HLA-DR expression on neutrophils. A decline in neutrophil HLA-DR expression was observed in those animals that would later relapse and succumb to disease; likewise, elevated circulating IFN-γ was detectable and indicative of relapse up to three days prior to death. At post-mortem, a reduced proportion of neutrophils in the blood was the only indicator of fatal disease. Minor immunological changes were observed between those animals that succumbed, recovered and later relapsed and those that survived. For example, there was a somewhat increased proportion of CD69+ CD8+ T-cells and decreased expression of CD40, CD16 and CD64 on macrophages. Interestingly, whereas neutrophil influx into the lung was a feature of those animals that received the placebo, there was no evidence for this in animals that received treatment and later relapsed. Akin to the situation in humans (Jenjaroen et al., 2015; Nithichanon et al., 2018), there was evidence of T-cell activation (indicated by expansion of the cytotoxic T-cell proportion and expression of CD16 and CD69) in animals that survived until the study end. The population of γδ T-cells was also expanded in survivors, providing additional evidence to support an important role for this cell type in the response to infection (Haque et al., 2006; Andreu-Ballester et al., 2013; Laws et al., 2013; Kronsteiner et al., 2019). Notably, a re-stimulation assay of splenic T-cells taken from those animals that survived revealed enhanced IFN-γ production compared with the negative control (Nelson et al., 2022a). In those animals that survived to the study end, high antibody titers were observed. Yet the relative protective value of the humoral response in humans is limited, despite the importance of vaccine-induced humoral immunity having been demonstrated in animal studies (Burtnick et al., 2018; Khakhum et al., 2019; Chaichana et al., 2020; Chaichana et al., 2021).

In summary, the common marmoset model of melioidosis has been well characterized and shown to recapitulate human disease and exhibit a higher degree of similarity to human disease compared with other animal models. It will no doubt have value in the evaluation and licensure of medical countermeasures.

### 3.4 Hepatitis C virus

Viral hepatitis, broadly defined as inflammation of the liver caused by a virus, represents a major health care burden worldwide (Estes et al., 2018; Jefferies et al., 2018). The hepatotropic viruses (types A to E) are the most important and common cause of hepatitis, with types B and C being most prevalent globally (Lim et al., 2020; Castaneda et al., 2021). Infection occurs either via ingestion of contaminated food or water (types A and E) or by contact with infected bodily fluids, i.e., blood (types B, C and D) (Loader et al., 2019). Hepatitis B can be transmitted from mother to baby at birth (Loader et al., 2019). Hepatitis A and D is typically acute and self-limiting, whereas types B, C and E can establish chronic disease (Loader et al., 2019; Castaneda et al., 2021). Chronic viral hepatitis is the leading cause of liver cirrhosis and hepatocellular carcinoma (Lin et al., 2014).

Tissue tropism of the phylogenetically unrelated hepatitis viruses for differentiated hepatocytes may explain the narrow range of susceptible hosts, namely humans and NHPs (Pfaender et al., 2014). Consequently, much of our knowledge of human viral hepatitis has stemmed from NHP models of infection. The proceeding discussion will focus on animal models of hepatitis C virus (and the closely related species GB virus B; the advantages and disadvantages of which are presented in **Table 6**) specifically. For reviews of animal models of the other hepatitis viruses, see (Purcell and Emerson, 2001; Manickam and Reeves, 2014; Protzer, 2017; Guo et al., 2018; Burwitz et al., 2020; Liu et al., 2021; Zhang et al., 2021).

**Table 6:** Marmoset and alternative animal models of hepatitis C virus (HCV) infection.

Marmoset model of hepatitis C virus infection			
Advantages	Disadvantages	Reference	
Cheaper and easier to breed in captivity Susceptible to GBV-B Infection rate and severity of acute infection similar to that in humans Acute viremia similar to that in chimpanzee Chronic, progressive disease similar to human HCV Acute disease exacerbation associated with chronic hepatitis Persistent infection established using HCV chimera Production of interferon-γ coincides with reduction of viral load Virus-specific T cells found predominately in the liver	Not susceptible to infection with HCV; studies rely on use of monkey-tropic viruses Infection may be acute or chronic depending on host Little characterization of immune response to infection, particularly between acute and chronic infection Humoral response to HCV infection requires further investigation Existence of mechanisms of T cell memory require further investigation	(Lanford et al., 2003; Bright et al., 2004; Jacob et al., 2004; Woollard et al., 2008; Weatherford et al., 2009; Iwasaki et al., 2011; Manickam et al., 2016)	

Alternative animal models of hepatitis C virus infection			
Model	Advantages	Disadvantages	Reference
Chimpanzee	First animal model for HCV infection Best characterized model of HCV infection *In vivo* virus replication Viremia Development of anti-HCV antibodies Elevated serum liver enzymes and necro-inflammatory changes in liver 60% of animals develop chronic disease	Natural course of infection different from that in humans Low availability of animals High costs Ethical concerns Disease course is significantly attenuated compared with human disease Limited availability of immunological reagents and tools	(Alter et al., 1978; Fernandez et al., 2004; Folgori et al., 2006; Puig et al., 2006; Bukh et al., 2008; Houghton, 2009; Manickam and Reeves, 2014; Pfaender et al., 2014)
Tamarins	Surrogate model of HCV infection Susceptible to experimental infection with GBV-B Persistent viremia Appearance of antiviral antibodies Induction of hepatitis Produces HCV-like disease Study of immune response associated with acute viral clearance	Surrogate model of HCV infection Disease is typically acute and self-resolving Failure to establish long-term or chronic viral persistence Not useful for vaccine development Difficult and costly to breed Limited availability of immunological reagents and tools	(Deinhardt et al., 1967; Beames et al., 2000; Beames et al., 2001; Lanford et al., 2003; Martin et al., 2003; Nam et al., 2004; Ishii et al., 2007; Takikawa et al., 2010; Iwasaki et al., 2011; Dale et al., 2020)
Tree Shrew	Susceptible to infection with HCV Persistent liver infection with some histological indications of liver disease Used in metabolomics studies to identify biomarkers of HCV infection Intermittent viremia and serum antibodies	Transient, self-resolving infection Intermittent viremia only if immunosuppressed Limited viral replication Limited availability of immunological reagents and tools	(Xie et al., 1998; Amako et al., 2010; Sun et al., 2013; Manickam and Reeves, 2014; Feng et al., 2017)
Mice	Can be manipulated to transgenically express individual or combinations of HCV gene products Transgenic mice useful for study of intrahepatic adaptive immune response Lots of well characterized strains, each with their own pros and cons Useful for antiviral drug evaluation Useful for immunization and challenge studies	Naturally resistant to HCV infection Disease severity is strain-specific Caveats associated with use of transgenic animals, e.g., failure to establish inflammatory milieu that is established during infection Chimeric mice are immunodeficient and thus are not useful for studies of HCV pathogenesis Lack of progressive liver pathology	(Galun et al., 1995; Mercer et al., 2001; Meuleman et al., 2005; Flint et al., 2006; Yang et al., 2008; Ploss et al., 2009; Bissig et al., 2010; Bitzegeio et al., 2010; Washburn et al., 2011; Anggakusuma et al., 2014; Hartlage et al., 2019)

Of all hepatitis viruses, hepatitis C virus (HCV) has the most restricted host range, capable of producing infection in humans and chimpanzees only (Folgori et al., 2006; Puig et al., 2006). As such, the majority of early studies of hepatitis C relied almost exclusively on chimpanzees, giving rise to first generation vaccines and a number of novel therapeutics. However, the search for alternative animal models of hepatitis C was fueled by increasing costs and ethical concerns surrounding the use of chimpanzees in biomedical research. Studies of the closely related GB virus B (Deinhardt et al., 1967), which infects new-world primates and produces disease similar to that caused by HCV in humans, were fundamental in expanding both the number and availability of alternative animal models.

#### 3.4.1 Common marmoset model of viral hepatitis C

The search for a more robust animal model of human HCV infection, particularly one permitting testing of vaccine efficacy, is important and remains a pressing unmet need in hepatitis C research. Whilst highly effective treatments for HCV infection exist, these are often prohibitively expensive and, consequently, are unavailable to those most at-risk individuals (Etzion and Ghany, 2015; Chahal et al., 2016). The development of preventative measures (like vaccines) is therefore key.

Development of a surrogate common marmoset model (Parks et al., 1969; Lanford et al., 2003; Bright et al., 2004; Kyuregyan et al., 2005; Haqshenas et al., 2007) of human HCV infection (with the NHP-specific GBV-B and, later, HCV chimera) followed earlier studies performed in tamarins (Deinhardt et al., 1967; Beames et al., 2000; Beames et al., 2001) which, compared to marmosets, are difficult and costly to breed in captivity. Though tamarins are susceptible to GBV-B infection, the utility of the tamarin model (and indeed monkey models more generally) of HCV infection was highly debated given the inability to establish chronic infection, a hallmark of human HCV infection (Lanford et al., 2003; Weatherford et al., 2009). The usefulness of the tamarin model was also limited by the availability of animals (Weatherford et al., 2009). Early studies in the marmoset revealed the susceptibility of the species to GBV-B infection, with animals developing acute viraemia (albeit to a lower level compared with that seen in tamarins) (Parks et al., 1969; Lanford et al., 2003; Bright et al., 2004). Interestingly, the level of viraemia in the marmoset was similar to that seen in chimpanzees (10^7^ copies/mL or less) which have been shown to develop persistent infections (Fernandez et al., 2004; Bukh et al., 2008). Thus, it has been suggested that lower viral loads in the acute phase of the infection may actually support viral persistence and the development of chronic inflammation (Iwasaki et al., 2011). Indeed, Iwasaki and colleagues were the first to show that infection of the marmoset with GBV-B produced a chronic and progressive disease similar to human hepatitis C, as indicated by fibrosis and recurrent increases of the liver enzyme alanine transaminase (ALT) (Iwasaki et al., 2011). Further, one marmoset experienced piecemeal necrosis and elevated ALT levels four years post-infection, indicative of an acute exacerbation associated with chronic hepatitis (Iwasaki et al., 2011), itself a feature of human viral hepatitis (Perrillo, 1997). Notably, marmosets infected with GBV-B were shown to exhibit two distinct phenotypes: susceptible and partially resistant (Weatherford et al., 2009). In contrast, HCV chimera (carrying core, E1, E2 and p7 structural proteins of HCV) causes persistent infection in marmosets (Li et al., 2014b). Since long-term viral persistence was established in animals with lower viral loads during the acute phase on infection (i.e., within the first 2 weeks post-infection), it seems reasonable to conclude that animals with the partially-resistant phenotype (where viral growth is restricted) will support the development of chronic infection. Viral persistence in those animals with lower viral loads may be the result of diminished early antiviral immune responses (Iwasaki et al., 2011). Data concerning the innate and adaptive immune response to infection in animals exhibiting acute disease compared with those that progress to develop chronic disease are still lacking and will prove critical in deciphering the mechanisms responsible for the establishment of chronic infection.

The induction of type I interferons represents one of the first responses to infection with HCV. HCV utilizes a NS3/4A protease to inactivate these early antiviral responses, possibly leading to viral persistence (Kaukinen et al., 2006). An interferon-inactivating NS3/4A protease is also present in GBV-B (Li et al., 2014b). In humans and chimpanzees, both CD4+ and CD8+ T cells play an important role in the response to HCV infection (Cooper et al., 1999; Lechner et al., 2000; Day et al., 2002; Woollard et al., 2003). The generation of virus-specific T cells that recognize multiple viral epitopes is crucial for viral clearance. Indeed, the accumulation of HCV-specific CD4+ and CD8+ T cells (recognizing multiple viral epitopes) in the liver is associated with acute resolving infection (He et al., 1999; Grabowska et al., 2001; Woollard et al., 2008). Conversely, a weaker T cell response against a limited number of viral epitopes is associated with viral persistence and chronic disease (Woollard et al., 2008). In the marmoset, IFN-γ production was first detectable five weeks post-infection, coinciding with a 1000-fold reduction in viral load (Woollard et al., 2008). A T cell response against NS3/N54A epitope (but no other viral epitope) was observed predominantly in the liver at week seven post-infection, coinciding with the clearance of viraemia (Woollard et al., 2008). At this point, virus-specific T cells appear in peripheral blood (Woollard et al., 2008). Akin to the situation in humans and chimpanzees, virus-specific T cells are present in higher frequencies in the liver than in the blood, suggesting the accumulation of T cells in the liver at the site of viral replication (He et al., 1999; Grabowska et al., 2001; Woollard et al., 2008). It is currently unclear whether the anti-HCV adaptive immune response is mediated by CD4+ or CD8+ T cells. Recently, the role of regulatory T cells (Tregs) in the response to HCV infection has gained increasing attention. Tregs, a unique type of CD4+ T cell with suppressor functions, are important in maintaining immune tolerance (Sakaguchi et al., 2008). In the context of an infection, Tregs can modulate effector T cell responses and, by inhibiting the anti-viral functions of specific T cells, may permit viral persistence (Boer et al., 2015; Liu et al., 2023). In chronically infected individuals, Treg populations are maintained, whereas the suppressor function of Tregs was diminished in individuals with acute resolving infection (Liu et al., 2023). The phenotype and role of Tregs in the marmoset is yet to be determined.

Another important aspect of the immune response against HCV is memory. In chimpanzees, virus-specific memory cells are essential for protection against reinfection (Grakoui et al., 2003; Shoukry et al., 2003). Marmosets were also protected from reinfection for several months after clearance of primary infection, pointing to the existence of virus-specific memory cells (Woollard et al., 2008). Consistently, T cell responses were both greater in magnitude and occurred faster following secondary infection, indicating recall of memory T cells (Bright et al., 2004; Woollard et al., 2008). In comparison to cell-mediated mechanisms of immunity, the humoral response to HCV infection is less well defined and requires further investigation.

In summary, the marmoset is susceptible to infection with both GBV-B and HCV chimeras and develops a hepatitis C-like disease, the pathology of which mirrors that of human HCV infection. Varying susceptibility phenotypes are likely genetically-determined, with some animals more likely to exhibit viral persistence and therefore chronic infection. In this sense, the marmoset may represent a valuable surrogate model of human hepatitis C.

## 4 Discussion

The common marmoset, a new-world primate, offers a number of advantages over the more traditional old-world primates; their small size, compact life-span and reduced husbandry costs are particularly notable, especially in the context of high containment research where their small size makes them both easier and safer to house. Their evolutionary proximity to humans makes them a more accurate and representative model of human disease compared to the more frequently used murine models. Critically, demonstration of the efficacy of medical countermeasures in a representative animal model is central to obtaining licensure under the FDA animal rule. Taken together, the marmoset represents an attractive alternative animal model. Further research in this area with increased focus on the development of marmoset-specific immunological reagents and tools will undoubtedly increase the utility of the marmoset in all areas of biomedical research.

## Author contributions

IH: Investigation, Writing – original draft, Writing – review & editing. TL: Conceptualization, Writing – review & editing. MN: Writing – review & editing.
